# A brief review of motor imagery and bimanual coordination

**DOI:** 10.3389/fnhum.2022.1037410

**Published:** 2022-11-11

**Authors:** Helene M. Sisti, Annika Beebe, Mercedes Bishop, Elias Gabrielsson

**Affiliations:** Department of Psychology, Norwich University, Northfield, VT, United States

**Keywords:** motor imagery (MI), bimanual coordination, brain computer interface (BCI), neurorehabilitation, motor control, EEG, neuroplasticity, learning

## Abstract

Motor imagery is increasingly being used in clinical settings, such as in neurorehabilitation and brain computer interface (BCI). In stroke, patients lose upper limb function and must re-learn bimanual coordination skills necessary for the activities of daily living. Physiotherapists integrate motor imagery with physical rehabilitation to accelerate recovery. In BCIs, users are often asked to imagine a movement, often with sparse instructions. The EEG pattern that coincides with this cognitive task is captured, then used to execute an external command, such as operating a neuroprosthetic device. As such, BCIs are dependent on the efficient and reliable interpretation of motor imagery. While motor imagery improves patient outcome and informs BCI research, the cognitive and neurophysiological mechanisms which underlie it are not clear. Certain types of motor imagery techniques are more effective than others. For instance, focusing on kinesthetic cues and adopting a first-person perspective are more effective than focusing on visual cues and adopting a third-person perspective. As motor imagery becomes more dominant in neurorehabilitation and BCIs, it is important to elucidate what makes these techniques effective. The purpose of this review is to examine the research to date that focuses on both motor imagery and bimanual coordination. An assessment of current research on these two themes may serve as a useful platform for scientists and clinicians seeking to use motor imagery to help improve bimanual coordination, either through augmenting physical therapy or developing more effective BCIs.

## Introduction

The rationale for this brief review was borne out of two observations: (1) brain computer interface (BCI) applications depend largely on motor imagery (Wolpaw and Wolpaw, 2012; McFarland and Wolpaw, 2018) (2) patient outcome can be improved when physical therapy is augmented with motor imagery (Mulder, 2007; Deutsch et al., 2012). Motor imagery is defined as the mental simulation of a movement in the absence of any overt movement (Decety, 1996; Jeannerod, 1999; Neuper et al., 2005). While the benefits of motor imagery have been recognized by athletes for decades, its advantages in clinical and rehabilitative settings, as well as its integration with BCI, is much more recent (Warner and McNeill, 1988; de Vries and Mulder, 2007). In BCI, individuals learn to control an external device, such as a neuroprosthetic device or more commonly, a computer cursor, by regulating their brain activity. Early demonstrations of this type of control were observed when individuals modulated their mu rhythm in the sensorimotor cortex (Wolpaw et al., 1991; McFarland et al., 2000; Neuper et al., 2009). More recent studies have demonstrated that BCI control is not limited to the mu rhythm, comprised of alpha and beta frequencies, but also occurs in several other ranges, including delta, theta, (Babiloni et al., 2017) and gamma rhythms (Babiloni et al., 2016). When a user learns to replicate this pattern, and conversely, the BCI accurately extracts and classifies the signature wave properties, then the individual acquires BCI control (McFarland and Wolpaw, 2018). This phenomenon requires multiple practice sessions to develop and is not present in all users (Vidaurre and Blankertz, 2010; Lotte and Jeunet, 2015; Thompson, 2019). It is important to acknowledge that movement execution and observation, which are closely related to motor imagery, have a complex underpinning. The neural dynamics that underlie movement execution and action observation overlap, and while they are highly relevant, they are beyond the scope of this brief review.

The majority of research in BCI has grown out of the machine learning perspective, i.e., developing computational algorithms that reliably interpret EEG signals (Aricò et al., 2018; McFarland and Wolpaw, 2018), while much less research has been dedicated to the nature of the user’s cognitive state. Further, stroke, and other neurodegenerative diseases such as multiple sclerosis, typically result in the loss of bimanual coordination. Many of the activities of daily living depend on successful bimanual coordination, such as buttoning a shirt or opening a tube of toothpaste. Therefore, a greater understanding of the use of motor imagery in bimanual coordination skills may help accelerate patient recovery and inform future research. The aim of this review is to bring together two themes, motor imagery and bimanual coordination, that are not often considered together, and to examine them in the context of their capacity to enhance neurorehabilitation and BCI development.

## Review criteria

This review was conducted using two scientific databases, PubMed and Scopus, with the keywords, motor imagery AND bimanual coordination. We adopted ‘*motor* imagery’ in favor or ‘*mental* imagery’ because the former is specific to movement and motor skills (Collet and Guillot, 2010), whereas the latter is a broader term that includes general cognitive tasks and does not necessarily require any movement. For example, mental rotation of a three-dimensional object, memory retrieval, and mental arithmetic would not be categorized as motor imagery. Bimanual coordination includes a diverse range of skills which depend upon simultaneous use of the left and right hands (Ivry et al., 2004; Swinnen and Wenderoth, 2004). The PubMed database retrieved 11 results and Scopus retrieved 17. All of PubMed’s results were duplicates of Scopus. Studies that focused on gait abnormalities in diseased populations (Vercruysse et al., 2012) or included TMS (Levin et al., 2004; Wang et al., 2015, 2016) were excluded.

## Movement topology

Bimanual coordination tasks range in complexity from those as simple as clapping, which an infant performs almost reflexively, to others that require a lifetime of practice, such as a concert pianist performing a difficult sonata. In the 16 research articles, the types of bimanual movements tested ranged from simple, laboratory tasks, such as bimanual sequence learning (Debarnot et al., 2012) and finger-thumb opposition task (Nair et al., 2003) to those high in ecological validity, such as tying shoelaces, buttoning a shirt (Szameitat et al., 2012) or mimicking piano playing (Riquelme-Ros et al., 2020). The simultaneous circle and line drawing task was used across a large range of ages and developmental stages (Piedimonte et al., 2014) in autistic spectrum conditions (Piedimonte et al., 2018) and in stroke patients (Morioka et al., 2019). The simultaneous circle-line drawing task is used as a method for investigating the bimanual coupling effect (Franz et al., 1991). That is, when a person attempts to draw a circle with one hand and a line with the other, the line starts to resemble a circle. The extent to which the linear trajectory takes the shape of a circle can be quantified using the ‘ovalization index’ (OI) (Garbarini et al., 2012; Piedimonte et al., 2014). This OI is thereby a quantitative measure of the degree of neural cross-talk. In other words, as the line being drawn with one hand, starts to resemble the circle being drawn by the other (or vice cersa), interference across the two hemispheres can be inferred (Franz et al., 1991; Garbarini et al., 2012; Piedimonte et al., 2014). This model was used in healthy (Piedimonte et al., 2014), autistic (Piedimonte et al., 2018), and stroke patients (Morioka et al., 2019). Morioka et al. (2019) noted that use-dependent plasticity is an important factor to consider in stroke patients. When movement execution becomes difficult or impossible due to a brain lesion, usually a contralateral one, the patient may stop even thinking about the movement. This type of motor neglect could contribute to a decline in motor representation. By asking patients to draw a line or circle with a paralyzed limb, some degree of motor planning was occurring. Therefore, even if movement execution was not observed, the motor representation was presumably being reactivated (Morioka et al., 2019). In their study, King et al. (2022) used a desktop pedal exerciser designed for the hands. They noted that bilateral arm training for the use of motor imagery in BCI is receiving increased attention because unlike unimanual motion, it enables (a) motor cortex disinhibition of the injured hemisphere, (b) enhanced paths from the contralesional region to recruitment, and (c) upregulation of descending motor neuron commands to propriospinal neurons (Whitall et al., 2011; Lee et al., 2016).

It is important to consider seminal research on bimanual coordination constraints. Haken et al. (1985) compared left and right hand movements to a coupled non-linear oscillator. In so doing, they developed what came to be known as the HKB model, which could make robust predictions concerning a range of bimanual coordination patterns (for full review, see Kelso, 2021). One of the implications of this model was that it brought to light the importance of movement topology (McGinnis and Newell, 1982; Schmidt and Lee, 2018). Briefly, when the left and right hands move in a symmetric or mirror-like pattern along the midline, they are easier to perform than when they are required to move in the same direction, i.e., in a parallel, asymmetric pattern (Swinnen, 2002). Further, the model accurately predicted that if individuals were asked to perform the less favorable, asymmetric movements at faster and faster rates, then bimanual coordination would be compromised; movements would start to transition to the intrinsically favorable mirror symmetric patterns (Swinnen and Wenderoth, 2004). Notably, bimanual coordination movements that were reliably predicted using *discrete* bimanual skills, such as finger tapping, could not be uniformly applied to those that required *continuous* bimanual skills, such as dial rotation (Sisti et al., 2011). For instance, in finger-tapping, some combinations can be learned at a faster rate others (Summers, 2002); integer ratios, where two taps of the hand occur for every one tap of the other (2:1) are easier to learn compared with non-integer ratios, three taps of one hand for every two taps of the other hand (3:2). However, when subjects are asked to perform a similar combination but with a continuous motor skill instead of a discrete one, this difference disappears and the relative velocity between the two hands becomes the predictive parameter (Sisti et al., 2011). When shifting from behavioral and musculoskeletal perspectives to one that is neurophysiological, the importance of movement topology is especially clear. That is, movement direction is uniquely encoded by neuronal populations (Georgopoulos et al., 1986). Therefore, there is both behavioral and neuronal evidence for the importance of considering movement topology in the context of developing BCI systems and neurorehabilitation strategies that enhance bimanual coordination.

## Motor imagery and Bernstein’s degree of freedom problem

Dahm and Rieger (2016a) point out that a variety of motor coordination constraints, such as Fitts’ Law, are also present in motor imagery (Fitts, 1954; Cerritelli et al., 2011; Dahm and Rieger, 2016a). They included three motor imagery conditions of a bimanual coordination task in order to determine the extent of cognitive constraints (Dahm and Rieger, 2016a). The study built on accumulating evidence for the role of cognitive and perceptual constraints in bimanual coordination (Mechsner et al., 2001). Briefly, participants learned target reaching tasks in which congruent and incongruent targets (X to O, vs. O to O) were included and required both symmetric and parallel movements to perform successfully. Both real and imagined movements were timed, and the three experimental conditions included were based on the phase of movement, i.e., initiation, termination, and the entire duration of the movement (Dahm and Rieger, 2016a). Participants engaged in kinesthetic imagery. Ease of imagery, vividness of imagery and concentration were all assessed using a Likert scale. It was noted that task-dependent processes occur in which several types of motor imagery may be differentially engaged. For instance, while drawing is a unimanual motor task, it relies heavily on visual cues, however, movements such as rowing or reaching may be more dependent on kinesthetic cues (Dahm and Rieger, 2016a). Further, components of the movement, such as initiation and termination, are important considerations. In another study, they examined whether bimanual coordination constraints are present in motor imagery (Dahm and Rieger, 2016b). They used three tasks to compare different versions of the mental chronometry paradigm, in which the time it takes to complete a motor imagery task is measured. The key finding was that bimanual coordination constraints were also present in motor imagery, i.e., imagined symmetric movements resulted in shorter inter-response intervals and greater accuracy than parallel movements (Dahm and Rieger, 2016b).

Szameitat et al. (2012) also included a kinesthetic first person perspective. Further, they instructed participants to “engage intensely” and with “high frequency” for the duration of a set time trial. Self-reported assessment of how vividly participants could imagine the movement was completed using a 7-point Likert scale. Szameitat et al. (2012) captured potential movement during imagery by having participants hold a force sensitive grip in each hand. Interestingly, the only condition that detected a significant increase from baseline was the most difficult one, i.e., imagination of a complex movement using the non-dominant hand.

The Russian neurophysiologist, Nikolai Bernstein (1967) first described what would come to be known as the degrees of freedom problem (1967). That is, redundant solutions exist for any given motor skill. When Bernstein’s degrees of freedom problem is considered in the context of motor imagery, redundant solutions multiply. In addition, there is the obvious issue that while behavior is directly observable, cognition is only indirectly observed. This highlights the need for specifying type of motor imagery, explicit *a priori* instructions as well as qualitative and quantitative *post hoc* assessments. The majority of papers reviewed included the favorable kinesthetic, first-person perspective, which emphasizes internal, proprioceptive sensations (see Table 1).

**TABLE 1:** A subset of articles from the literature review that focused on neurologically health adults.

References	Movement type	Motor imagery	Neuro-imaging technique	Population studied	Key points
Riquelme-Ros et al., 2020	Drumming of the fingers, swinging wrist up and down; mimicked piano playing – Imagined only	Not specified	EEG	Expert piano players (*n* = 4; 2 males, 2 females, mean 24.5 years) vs. controls (*n* = 4; 2 males, 2 females, mean 32.75 years)	-The Common Spatial Patterns (CSP) machine learning algorithm was applied to *feature extraction* and Linear Discriminant Analysis (LDA) machine learning algorithm was applied to *classification* using 8 electrode cap. -Expert pianists achieved higher level of BCI control (75%) compared with non- pianists (63%) using motor imagery.
Dahm and Rieger, 2016a	Target task including reaching in response to a cue; reaction time, movement time, and movement preparation time were captured	Kinesthetic imagery at each phase of the movement, pressing and holding the start button, reaching for the target and pressing the target	Cognition and behavior only	Healthy adults (*n* = 23, mean age 24.5 years)	-Bimanual coordination constraints are present in motor imagery. -Functional equivalence between real and imagined movement is present even for short reaction times to stimuli. -Task-dependent differences occur in motor imagery; some rely more heavily on kinesthetic representation whereas others rely more heavily on a visual representation. -Different subcomponents of movement are not equally vivid during motor imagery.
Sallard et al., 2014	Unimanual and bimanual finger tapping	Not specified	EEG	Young (*n* = 29; 14 males, 15 females; age range 19-29 years) vs. Old (*n* = 27; healthy adults, 12 males 15 females; age range 60–83)	-Main effect of tapping condition in both low and high beta bands, with lower power in bimanual than unimanual in both age groups. -Bimanual advantage is absent in the elderly; Motor imagery was not included in the methods, rather in the interpretation of the results. -Elderly may rely more on visual imagery compared with kinesthetic imagery due to reduced kinesthetic reafferences.
Debarnot et al., 2012	Simple and complex unimanual and bimanual finger sequence learning	Kinesthetic, internal visual	Cognition and behavior only	Healthy adults (*n* = 48, gender not reported, mean age 27.8 years	-Sleep following Motor Imagery training improved performance compared to same passage of time with no sleep (daytime interval). -Strongest performance gains were obtained for the most challenging task; this finding illustrates that similar to learning a physical motor skill, sleep enhances memory consolidation of MI, and the effect is enhanced as the difficulty of the skill increases.
Szameitat et al., 2012	Everyday tasks such as tying shoelaces, buttoning a shirt	Kinesthetic first person	fMRI	Neurologically healthy adults (*n* = 17, 6 male, 11 female, aged 19-31; mean 22 years)	-High in ecological validity; results consistent with laboratory models of bimanual coordination. -Detected changes in functional connectivity within and between cerebral hemispheres. -Highlighted importance of multivariate network analysis rather than region-of-interest approach.
Nair et al., 2003	Unimanual and bimanual finger to thumb opposition task	Not specified	fMRI	Neurologically healthy adults (*n* = 8; 3 males and 5 females; ages 25–40)	-Sensorimotor cortex, supplementary motor area (SMA), superior parietal lobule and cerebellum were identified when the tasks involved both planning and execution. -Cerebellar activity present during actual, but not imagined, movement; thereby supporting motor control theory that the cerebellum monitors cortical output and provides corrective information to the motor cortex primarily during physical, executed movement. -Subjects reported that imagining the bimanual sequence movement was the most difficult of the tasks performed.

Clinical populations, such as Parkinson’s disease, cerebral palsy or autistic spectrum disorder, are beyond the scope of this brief review.

## Brain mapping approaches: Region of interest and functional connectivity

The first demonstration of the neuroanatomical correlates of imagined bimanual coordination skills was reported by Szameitat et al. (2012). Their prediction, and substantiation, that the neural activity of a specific cortical region of interest would not be selectively increased, rather connectivity would change, was built on the previous research of Puttemans et al. (2005). They reported that when skills are learned to the point that they become automatic, they are said to be overlearned, and in this case the neuroanatomical correlates are *sub*cortical, and not cortical. In the early stages when attentional resources and cognitive demands are high, cortical regions are engaged; however during later stages of learning, cortical activation dissipates and subcortical activations become dominant (Puttemans et al., 2005). Szameitat et al. (2012) found changes in functional found changes in functional connectivity between parietal and premotor areas within and between hemispheres. This was in contrast to the lack of effect of any change in neural activation of a specific cortical region. This distinction is noteworthy because EEG captures cortical changes, while activity changes in subcortical structures depend on how well the “source localization” problem is addressed.

Coordinating the left and right hands depends on the primary and premotor cortices (M1 and PMC) and supplementary motor area (SMA) in the cortex, the cerebellum, and subcortically, the cingulate motor area and basal ganglia (Swinnen and Wenderoth, 2004; Jantzen et al., 2008; Rueda-Delgado et al., 2014). These areas are also active in unimanual movements (Cabibel et al., 2020). As task demands increase, the network extends beyond these regions to encompass prefrontal, parietal, and temporal areas (Swinnen and Wenderoth, 2004; Rueda-Delgado et al., 2014). While contralateral motor control has long been established, recent studies have highlighted the important contributions of ipsilateral motor control as well (Bundy and Leuthardt, 2019). Several studies have shown that the sensorimotor areas of the ipsilateral hemisphere are also activated (Bundy and Leuthardt, 2019), though its role is not yet clear. Some sustain that the ipsilateral activation contributes to the planning and execution of contralateral limb movements as the kinematic and movement parameters of the contralateral limb can be decoded from signals recorded in the ipsilateral hemisphere in monkeys (Ames and Churchland, 2019) and in humans (Bundy et al., 2018). Furthermore, the two hemispheres seem to have different roles in terms of praxis, independently from handedness. In fact, lesions of the left hemisphere lead to apraxia, i.e., the impairments in the production of complex movement in the absence of muscle deficits, much more often than right hemisphere damages (Haaland, 2006). Notably, a study showed that the ipsilateral cortical representation of reaching arm movements strikingly differs between the left and right hemispheres (Merrick et al., 2022). Finally, Mancini and Mirabella (2021) found left dominance in reactive inhibitory control. Inhibitory control is a pillar of motor control (Mirabella, 2014), and very likely, implementing inhibitory control in BCI could greatly help in restoring naturalistic behaviors (Mirabella and Lebedev, 2017).

## Conclusion

-Developing effective BCI systems and neurorehabilitation strategies may depend on careful consideration of the type of bimanual movements individuals are being asked to learn.

-Skills that have been learned so well that they become automatic rely more heavily on subcortical structures and less so on cortical structures. EEG captures only surface cortical changes, and not subcortical ones. Although activity from deeper brain structures may be inferred as the “source localization” problem is resolved, EEG does not have the spatial resolution of fMRI. Therefore, movement topology and learning factors must be considered. For instance, the inclusion of more complex bimanual patterns may reveal cortical activation patterns that are distinct from simpler bimanual coordination patterns that require little training to learn.

-Assessing the vividness of a person’s motor imagery skills using both qualitative and quantitative measures, such as R-VMIQ and mental chronometry, may account for unexplained variance, thereby leading to more effective classifiers in BCI and better MI intervention strategies in clinical settings.

## Author contributions

HS conceived the topic, led the literature search, prepared the manuscript, and final table. AB, MB, and EG conducted the literature searches, retrieved full-text articles, and prepared earlier versions of the table. All authors contributed to the article and approved the submitted version.
